# Management of Traumatic Facial Soft Tissue Injuries: A Hospital-Based Retrospective Study

**DOI:** 10.7759/cureus.103070

**Published:** 2026-02-05

**Authors:** Spurthi S., G.S.R. Hareesh, Lakshmi Meena Jasti

**Affiliations:** 1 Plastic and Reconstructive Surgery, Government Medical College, Ongole, Ongole, IND; 2 General Surgery, Government Medical College, Ongole, Ongole, IND

**Keywords:** aesthetic restoration, facial trauma, flap reconstruction, soft-tissue injuries, wound management

## Abstract

Objective: This study aimed to evaluate the demographic distribution, injury sites, and management strategies for soft-tissue facial trauma at a tertiary-care center, highlighting the role of timely reconstruction in achieving optimal functional and aesthetic outcomes.

Methodology: A retrospective analysis was conducted at Government Medical College, Ongole, including patients presenting with isolated soft-tissue facial injuries between 2021 and 2023. Data on demographics, injury sites, wound types, and reconstructive modalities were collected and analyzed.

Results: Among the 50 patients included, 34 (68%) were male, and 16 (32%) were female. Flap reconstruction was performed in 16 (32%) patients, primary closure in 13 (26%), delayed primary closure in 12 (24%), conservative management in seven (14%), and skin grafting in two (4%) (χ^2^ = 14.56, p = 0.006). Among flap reconstructions, advancement flaps were the most frequently used, accounting for eight (50%) cases (χ^2^ = 8.92, p = 0.031).

Conclusion: Soft-tissue facial injuries were most commonly observed in young adult males, with road traffic accidents as the leading cause and lacerations as the predominant injury type. Flap reconstruction was the most frequently used management approach, particularly for complex defects, with local advancement flaps preferred. Timely and appropriate reconstruction played a key role in achieving satisfactory functional and aesthetic outcomes.

## Introduction

Facial trauma accounts for approximately 7% of all emergency department visits [1]. The severity of injury depends on the mechanism, direction, and velocity of impact, ranging from superficial soft-tissue damage to involvement of deeper structures. Because the face plays a central role in personal identity and social interaction, facial trauma can have significant functional and psychosocial consequences [2,3]. The primary goals of facial reconstruction are to restore symmetry, preserve function, and maintain aesthetic integrity, ideally during the initial surgical intervention [4,5]. Soft-tissue facial injuries may result from motor vehicle collisions, falls, assaults, occupational accidents, sports-related incidents, animal or human bites, burns, and, rarely, self-inflicted wounds.

This hospital-based retrospective study evaluates the epidemiology, injury patterns, and management outcomes of soft-tissue facial trauma treated at Government Medical College, Ongole, India, between 2023 and 2025. The objectives were to assess the distribution of injury sites, analyze demographic characteristics of affected patients, and describe the reconstructive techniques employed. The study also emphasizes the importance of early, appropriate reconstruction to achieve optimal functional and aesthetic outcomes.

## Materials and methods

Study design and setting

This hospital-based retrospective observational study was conducted in the Departments of Plastic Surgery and General Surgery at Government Medical College, Ongole, India. Patients managed for facial soft-tissue trauma over a 30-month period, from January 2021 to June 2023, were included in the study. Medical records and operative logs were reviewed to analyze epidemiological characteristics, injury patterns, etiologies, and reconstructive management of facial soft-tissue injuries.

Study population and sampling technique

A consecutive sampling technique was employed. All patients presenting with facial trauma during the study period were screened for eligibility. Of the 82 patients who presented with facial injuries, 50 met the predefined inclusion and exclusion criteria and were included in the final analysis. This approach ensured comprehensive inclusion of eligible cases and minimized selection bias inherent to retrospective studies.

Inclusion and exclusion criteria

Patients of all ages and both sexes presenting with isolated facial soft-tissue injuries, including lacerations, abrasions, avulsions, tissue-loss injuries, burns, and bite wounds, were included. Patients with associated facial bone fractures, intracranial injuries, or polytrauma requiring multidisciplinary management were excluded to maintain a homogeneous study population and to minimize confounding related to complex injuries. Patients with incomplete clinical documentation or inadequate follow-up records were also excluded.

Data collection and management

All patients were initially stabilized according to Advanced Trauma Life Support (ATLS) protocols [6]. Retrospective data were extracted from inpatient case records, operative notes, and outpatient follow-up charts using a structured data collection format. Demographic variables included age and sex, with age categorized into 0-10 years, 11-40 years, and >40 years for analysis. Injury-related variables comprised anatomical site of injury (scalp, eyelid, cheek, nose, and lip), etiology (road traffic accidents, falls, occupational injuries, and assault), and type of soft-tissue injury (abrasion, laceration, avulsion, and tissue loss).

A detailed clinical assessment documented wound size, depth, and involvement of facial aesthetic subunits. Clinical photographs obtained at presentation and during follow-up were used for documentation and outcome assessment. All data were anonymized before analysis to ensure patient confidentiality.

Wound management and reconstructive procedures

All wounds were managed according to standardized institutional protocols. Initial management included copious irrigation with sterile saline and conservative debridement of devitalized tissue. Treatment modality was individualized based on wound characteristics, extent of tissue loss, anatomical location, and anticipated functional and aesthetic outcomes. Management options included conservative treatment, primary closure, delayed primary closure, split-thickness skin grafting, and flap reconstruction.

For cases requiring flap coverage, the type of flap used (e.g., advancement, rotation, transposition, nasolabial, or forehead) was documented. Reconstructive decisions were guided by established principles of facial vascular anatomy and aesthetic unit reconstruction.

Statistical analysis

Statistical analysis was performed using IBM SPSS Statistics for Windows, Version 26 (Released 2018; IBM Corp., Armonk, New York, United States). Continuous variables were expressed as mean ± standard deviation, while categorical variables were summarized as frequencies and percentages. Associations between categorical variables, including age group, sex, injury site, etiology, type of injury, and reconstructive modality, were assessed using the chi-square (χ^2^) test. Post hoc pairwise comparisons were performed where applicable. A p-value <0.05 was considered statistically significant. Results were presented in tables and graphical formats to facilitate interpretation.

Ethical considerations

Ethical approval was obtained from the Institutional Ethics Committee of Government Medical College, Ongole (IEC/GMC-OGL/197/2024). The study was conducted in accordance with the Declaration of Helsinki. As this was a retrospective study, the ethics committee waived informed consent, and strict confidentiality of patient data was maintained throughout.

## Results

The study population included 34 (68%) males and 16 (32%) females. Age distribution showed that 15 (30%) participants were 0-10 years, 26 (52%) were 11-40 years, and nine (18%) were over 40 years, with differences in both gender and age groups reaching statistical significance (χ^2^ = 6.48, p = 0.011 and χ^2^ = 9.72, p = 0.008, respectively) (Table 1).

**Table 1:** Distribution of patients according to gender and age group (n = 50) * indicates a statistically significant difference. A p-value < 0.05 was considered statistically significant.

Variable	Category	N (%)	Chi-square test	Degree of freedom (df)	p-value
Gender	Male	34 (68)	6.48	1	0.011*
	Female	16 (32)			
Age group (years)	0-10	15 (30)	9.72	2	0.008*
	11-40	26 (52)			
	>40	9 (18)			

Lesions were most frequently observed on the scalp in participants aged 11-40 years (12, 24%) and least often in the 0-10 years group (1, 2%). Other common lesion sites included the lip and nose among the 11-40 years group, while the eyelid and cheek were more evenly distributed across age groups. Overall, the differences in lesion distribution across age groups were statistically significant (χ^2^ = 12.84, p = 0.045), indicating that certain lesion sites were more prevalent in specific age groups (Table 2).

**Table 2:** Site-wise distribution of facial soft-tissue injuries by age group * indicates a statistically significant difference. A p-value < 0.05 was considered statistically significant.

Site	0-10 yrs N (%)	11-40 yrs N (%)	>40 yrs N (%)	Total	Chi-square test value	Degree of freedom (df)	p-value
Scalp	1 (2)	12 (24)	2 (4)	15	12.84	8	0.045*
Eyelid	2 (4)	4 (8)	4 (8)	10			
Cheek	5 (10)	5 (10)	1 (2)	11			
Nose	3 (6)	7 (14)	2 (4)	12			
Lip	4 (8)	8 (16)	1 (2)	13			

Table 3 illustrates the etiological distribution of facial soft-tissue injuries. Road traffic accidents were the leading cause, accounting for 28 (56%) cases, followed by falls (11, 22%), occupational injuries (6, 12%), and assault (5, 10%). The predominance of road traffic accidents was statistically significant (χ^2^ = 18.67, df = 3, p < 0.001).

**Table 3:** Distribution of causes of facial injuries in the study population Chi-square (χ^2^) = 18.67, df = 3, p < 0.001

Cause	N (%)
Road traffic accidents	28 (56)
Falls	11 (22)
Occupational injuries	6 (12)
Assault	5 (10)

Lacerations were the most common injury type, observed in 25 (50%) participants, followed by tissue loss in 10 (20%), avulsion in eight (16%), and abrasions in seven (14%). The distribution of injury types differed significantly among participants (χ^2^ = 10.23, p = 0.017), highlighting laceration as the predominant injury in this population (Table 4).

**Table 4:** Distribution of types of injury among the study population * indicates a statistically significant difference. A p-value < 0.05 was considered statistically significant.

Type of injury	N (%)	Chi-square test	Degree of freedom (df)	p-value
Abrasion	7 (14)	10.23	3	0.017*
Laceration	25 (50)			
Avulsion	8 (16)			
Tissue loss	10 (20)			

Among the study population, flap cover was the most frequently employed management method, observed in 16 (32%) participants, followed by primary closure in 13 (26%), delayed primary closure in 12 (24%), conservative management in seven (14%), and skin grafting in two (4%). The differences in the distribution of management methods were statistically significant (χ^2^ = 14.56, p = 0.006) (Table 5).

**Table 5:** Distribution of management methods used for facial injuries * indicates a statistically significant difference. A p-value < 0.05 was considered statistically significant.

Method	N (%)	Chi-sqaure test	Degree of freedom (df)	p-value
Conservative	7 (14)	14.56	4	0.006*
Primary closure	13 (26)			
Delayed primary closure	12 (24)			
Skin grafting	2 (4)			
Flap cover	16 (32)			

Table 6 shows the distribution of flap types among the 16 patients who underwent flap reconstruction. Advancement flaps were the most commonly used (8, 50%), followed by rotational flaps (4, 25%), transposition flaps (2, 12.5%), and nasolabial and forehead flaps (one each, 6.25%).

**Table 6:** Distribution of flap types used for reconstruction (n = 16)

Flap type	N (%)
Advancement flap	8 (50.0)
Rotational flap	4 (25.0)
Transposition flap	2 (12.5)
Nasolabial flap	1 (6.25)
Forehead flap	1 (6.25)

## Discussion

Soft-tissue facial injuries pose unique challenges because they often involve multiple aesthetic subunits and functionally critical structures, making restoration of both form and function essential [7,8]. In this study, males accounted for 68% of patients, likely reflecting greater exposure to road traffic accidents, occupational hazards, and outdoor activities. The most affected age group was 20-40 years, representing the socially and economically active segment of the population, consistent with previous reports [7,8].

These injuries vary widely, ranging from minor linear lacerations to extensive tissue destruction that can cause significant deformity. The incidence of soft-tissue involvement in maxillofacial trauma differs globally [1,9,10]. In this study, tissue-loss injuries occurred in 20% of cases, compared with 53.59% reported by Rehman et al. [11]. Eyelid injuries were observed in 20% of patients and scalp injuries in 30%, similar to the 29% reported by Gataa and Muassa [12] for severe soft-tissue, eye, and brain injuries. Findings from studies in India [13] and Nigeria [14] align with our results, reflecting comparable demographic and social contexts.

Analysis of anatomical distribution showed that patients aged 11-40 years sustained the highest number of injuries across all facial sites. Scalp injuries predominated in this age group, while cheek injuries were more common in children, and eyelid injuries were relatively frequent among older patients. Motor vehicle accidents were the leading cause of trauma, followed by falls, occupational injuries, and interpersonal violence, highlighting the impact of high-energy mechanisms in facial soft-tissue injuries.

Lacerations were the most common injury type, followed by tissue-loss and avulsion injuries, consistent with findings by Ong and Dudley [1]. Effective management relies on fundamental principles, including hemostasis, infection control, preservation of viable tissue, and tension-free wound closure. Regular wound irrigation, use of moist dressings, and minimal cautery were emphasized to optimize healing [15,16]. Antibiotics were reserved for contaminated wounds, bite injuries, and cases requiring delayed closure, in accordance with established recommendations [16,17].

A range of surgical techniques was employed for soft-tissue repair, including primary closure, conservative management, and local flap application. Wu [18] reported excellent aesthetic outcomes using fundamental techniques, while Hove et al. [19] and Roger et al. [20] applied Z-plasty and W-plasty, respectively, for facial injuries.

Reconstructive strategies were individualized based on wound characteristics, extent of tissue loss, and involvement of aesthetic units. Simple lacerations were managed with layered primary closure under local anesthesia [5], whereas complex defects required advanced reconstructive techniques. Flap reconstruction was the most commonly employed modality, followed by primary and delayed primary closure. Local flaps, particularly rotational flaps, were frequently used and provided satisfactory functional and aesthetic outcomes with minimal donor-site morbidity. The predominance of local flaps underscores the importance of early and appropriate reconstruction, consistent with Aveta and Casati, who reported improved outcomes with timely aesthetic reconstruction [2].

This study has certain limitations, including its retrospective design and relatively small sample size, which may limit generalizability. Prospective studies with larger populations and long-term follow-up, including objective assessment of aesthetic outcomes, are warranted to strengthen the evidence base.

## Conclusions

This hospital-based retrospective study demonstrates that isolated soft-tissue facial injuries predominantly affect young adult males, with road traffic accidents being the leading cause. The scalp, lips, and nose were the most commonly involved sites, and lacerations constituted the most frequent injury pattern. Management was individualized based on wound characteristics, with flap reconstruction emerging as the most widely employed modality, particularly for complex defects. At the same time, primary and delayed primary closure remained effective for selected cases. Among reconstructive options, local advancement flaps were most frequently used, reflecting their reliability and suitability for achieving favorable functional and aesthetic outcomes. Overall, the study emphasizes that timely evaluation and appropriate selection of reconstructive techniques are essential for optimal restoration of facial form and function in patients with soft-tissue facial trauma.

## References

[1] Ong TK, Dudley M (1999). Craniofacial trauma presenting at an adult accident and emergency department with an emphasis on soft-tissue injuries. Injury.

[2] Aveta A, Casati P (2008). Soft tissue injuries of the face: early aesthetic reconstruction in polytrauma patients. Ann Ital Chir.

[3] Jaiswal R, Pu LL (2013). Reconstruction after complex facial trauma: achieving optimal outcome through multiple contemporary surgeries. Ann Plast Surg.

[4] García-Gubern CF, Colon-Rolon L, Bond MC (2010). Essential concepts of wound management. Emerg Med Clin North Am.

[5] Wu PS, Beres A, Tashjian DB, Moriarty KP (2011). Primary repair of facial dog bite injuries in children. Pediatr Emerg Care.

[6] American College of Surgeons (2018). Advanced Trauma Life Support: ATLS® Student Course Manual. Surgeons.

[7] Kraissl CJ (1951). The selection of appropriate lines for elective surgical incisions. Plast Reconstr Surg (1946).

[8] Wilhelmi BJ, Blackwell SJ, Phillips LG (1999). Langer’s lines: to use or not to use. Plast Reconstr Surg.

[9] Key SJ, Thomas DW, Shepherd JP (1995). The management of soft tissue facial wounds. Br J Oral Maxillofac Surg.

[10] Gustilo RB, Anderson JT (1976). Prevention of infection in the treatment of one thousand and twenty-five open fractures of long bones: retrospective and prospective analyses. J Bone Joint Surg Am.

[11] Rehman B, Qiam ud Din, Ansari SR (2014). Soft tissue involvement in maxillofacial trauma and its financial impact on health resources. J Khyber Coll Dent.

[12] Gataa IS, Muassa QH (2011). Patterns of maxillofacial injuries caused by terrorist attacks in Iraq: retrospective study. Int J Oral Maxillofac Surg.

[13] Kapoor P, Kalra N (2012). A retrospective analysis of maxillofacial injuries in patients reporting to a tertiary care hospital in East Delhi. Int J Crit Illn Inj Sci.

[14] Adeyemo WL, Ladeinde AL, Ogunlewe MO, James O (2005). Trends and characteristics of oral and maxillofacial injuries in Nigeria: a review of the literature. Head Face Med.

[15] Heal CF, Banks JL, Lepper PD, Kontopantelis E, van Driel ML (2016). Topical antibiotics for preventing surgical site infection in wounds healing by primary intention. Cochrane Database Syst Rev.

[16] Sabatino F, Moskovitz JB (2013). Facial wound management. Emerg Med Clin North Am.

[17] Zide BM, Swift R (1998). How to block and tackle the face. Plast Reconstr Surg.

[18] Wu T (2006). Plastic surgery made easy - simple techniques for closing skin defects and improving cosmetic results. Aust Fam Physician.

[19] Hove CR, Williams EF 3rd, Rodgers BJ (2001). Z-plasty: a concise review. Facial Plast Surg.

[20] Rodgers BJ, Williams EF, Hove CR (2001). W-plasty and geometric broken line closure. Facial Plast Surg.

